# Domestic

**Published:** 1841-02-13

**Authors:** 


### DOMESTIC.

In the last number of the *New York Journal of Medicine and Surgery*, there is a capital paper on *the purulent opthalmia of Almshouses, by N. Morrell, Assistant Physician, Long Island Farms*. The article is too long to permit us to quote it entire, but we will endeavour to present, in a condensed form, all that it contains of practical importance. The contaminated atmosphere common to Hospitals, Schools, and Asylums, and particularly to Almshouses, where numbers are crowded together, and cleanliness is not attainable, is always deleterious, and particularly to those who are not habituated to it.

"In Hospitals it produces diarrhœa, almost without exception, among the pupils of medicine when they first commence visiting the wards. In most Almshouses, diarrhœa is perpetually endemic as well among adults as children. In boarding schools the more common effect is typhus fever, where the external atmosphere combines to excite the causes within. In asylums for children, diarrhœa and ophthalmia are of frequent occurrence, and frequently endemic; but ophthalmia is not confined to asylums attached to Poorhouses; it pervades many other institutions of a similar character.

"Since mucous or purulent ophthalmia has become so common, the means of prevention deserve the first consideration, which leads to the history of the disease in the New York Almshouse, an asylum at present for more than seven hundred children. In 1831, these children were provided for at Bellevue, where at the time, all the above mentioned causes were rife; and though it is supposed that contagion was first introduced, these causes kept it in fermentation, so that more than half the children there, (about four hundred,) were unfit for school; several became totally blind, others recovered with the loss of one eye.

"Under such circumstances the most experienced physicians were consulted, and finally employed. Under their direction and advice, especially under the direction of Dr. Cheesman, the treatment was improved, scarcely one lost both eyes, and few even one; still the number in the hospital did not greatly diminish. The farms were then purchased, and here Dr. Bushe took the direction. Under a pure ventilation the disease was not so destructive; but the cases attacked were not less numerous than before, and the hospital for ophthalmia still continued its usual quota. At this time, the 8th of January, 1833, the writer went to Blackwell's Island, and took charge of the nurseries at the farms."

Dr. Morrell had previously studied this disease, and while acknowledging its contagious character, and the predisposition to its developement which existed occasionally, conceived

"That the above named causes, acting on this hereditary predisposition, would produce in the first place, disease of the conjunctiva; that this, by rubbing, would turn to inflammation, and this terminate in a purulent discharge poisonous to the mucous membranes of a healthy child wherever applied, but especially to the mucous membrane of the eye, and thus generate an endemic disease, poisonous even at a distance, from a very simple form of inflammation in a very vitiated constitution.

"To prevent the disease therefore, the physician directed that the children be divided into squads; that the name and number of each squad be taken and marked on the beds and blankets used by each squad; that no bed or blanket used by one child should be appropriated to another without previous washing; that every child be supplied with a towel for itself, firmly sewed on every morning, to be used through the day and on the next morning, when every child was to wash either in separate water, or in water poured on his hands held over a tub, from another tub standing near, and filled with clean water; that the officer appointed to oversee the children, should be present at every ablution, and see that these directions were fully accomplished. In addition, the physician presented himself every morning, was frequently present during ablution, and in the dormitories at night, to see that his requisitions were fully answered: he also directed, and saw that his direction was performed, that the buildings which were not constructed for ventilation should be ventilated at night by the windows being left partially open, and that the dormitories should not be occupied for day rooms, or the day rooms for dormitories—thus allowing a tolerable opportunity for the purification of the air in each apartment during the absence of the children. He also directed that every thing washed should be washed in the most careful manner possible, and rendered perfectly clean. Now these appear to be regulations perfectly simple, and capable of being executed with ease. But the present writer found it very difficult, and could not procure the performance of all for more than a year after commencing; he did, however, finally procure their complete accomplishment, and had the gratification of seeing the disease gradually diminish year after year, till he has not now, the 15th of November, 1840, more than twenty out of seven hundred with ophthalmia, and these for the most part slight affections, while the others are as healthy as to their eyes, as any children in private families."

In the Deaf and Dumb Asylum the same precautions were productive of like effects. The author names the affection Almshouse Ophthalmia, because, in such institutions, the discharge, although mucous in the beginning, *commonly* becomes purulent. The signs of catarrhal ophthalmia through all its stages, up to the Egyptian or gonorrheal form, are so familiar that we omit their detail

At the period of the secretion of pus "the inflammation has already passed to new formation on the surface of the palpebral conjunctiva, but the pus is not secreted free enough to relieve the vessels underneath; the tumefaction continues and increases, while the pain becomes excruciating. At the junction of the seventh and fifth pair of nerves over the orbit, and also at the plexus underneath it, the pain is excessive; and from this pain the patient can obtain no relief without opium, unless the disease abate. In such cases, the writer divides the temporal artery, and lets the blood freely till the pain is relieved and the conjunctiva turns pale."

This is followed by wine of colchicum and emetics, as long as the patient can well bear to vomit, succeeded by a blister over the forehead and partly on the lids. If this fail, and photophobia still exists, with danger of sloughing of the cornea, he "takes his lancet, or a scalpel which is better, depresses the lower lid with the thumb of his left hand, while an assistant carefully raises the upper, at which moment the ball will turn up to avoid the light, and leave the point of reflection of the conjunctiva from the lid to the globe nearly exposed, and as near this point as possible, makes an incision completely through the conjunctiva to the ball for near half the circumference of the cornea. The blood flows freely, the chemosis subsides immediately, and danger to the eye is nearly past. He then continues the wine of colchicum in doses to suit the situation of the patient, and the wash of tepid milk and water till the ball is cleared off, and then applies the crystal of sulphate of copper to the lids until a cure is effected.

"In other cases, which are rare after the first two or three days of treatment, the swelling leaves the lids but remains in the conjunctiva of the ball, and as the writer believes, in the ball itself. Certainly the ball appears swollen, red, and protruding through the now attenuated lids, which cannot entirely close over it. It becomes terribly painful and as if ready to burst, which finally happens, notwithstanding any treatment known at present to the author; but fortunately such cases have always occurred in the writer's experience where only one eye was attacked, while the other remained unaffected throughout."

The passage from the acute to the chronic form of this disease, which latter consists in granulations of the conjunctiva, followed by a vascular keratitis is next considered, and the daily application of a crystal of sulphate of copper to the lids is enforced; but the author still recommends that the temporal artery be opened and permitted to bleed freely "*if occasion require.*"

"And when the internal surface of the lids has become tolerably smooth, he takes a cornea knife, depressing needle or even a lancet, and divides completely every vessel of the conjunctiva carrying red blood to the ulcers; leaves off all other treatment for a few days, then reapplies the sulphate of copper to the lids, and gives phosphorous internally. Under the use of these remedies the ulcers, no longer irritated by unnatural vessels or granulated lids, heal completely, and the cornea becomes nearly as transparent as ever. Indeed in all ulcerations of the cornea, it is beneficial to divide the vessels of the conjunctiva, if any pass into the ulcers; and some cases of opacity even, can be cured by such divisions."*

Dr. M. next treats of purulent opthalmia as modified by constitutional causes, but as the symptoms given by him do not differ from those of other writers on opthalmia, and are not so well arranged as the preceding portion of his paper, we omit them in order to present in full his plan of treatment.

"In addition to what has already been remarked, great care should be taken while treating opthalmia in Alms houses where there are frequently from ten to twenty confined in a room, that the matter from the eye, in the acute stage, be not communicated to another child, as this adds greatly to the production of relapses, and frequently to the severity of the acute stage. All in this stage should be confined to their beds, and great care be taken that the bedclothes of the one be not transferred to the other, because this is not a specific disease which passes a certain course and completes itself, but one liable to be repeated at every new infection, and to communicate inflammation to a healthy eye for months if the matter be introduced, precisely as the secretion from an old ulcer excoriates and inflames the surrounding skin, if it be at any time neglected. Hence the physician who hopes to banish the endemic prevalence of this disease from any institution in which he may find himself engaged, must follow it perpetually from the hospital to the school-room, and from the school-room to the dormitories, applying at every appearance of a recurring discharge his blue-stone, which he should carry perpetually in his pocket. He should turn the lids well out, and apply the crystal freely or otherwise, as the state of the eye may demand. Old hardened granulations should be well rubbed, while milder forms should be touched more gently. By steadily pursuing this course, the physician will neither need ointments, nor washes, nor cupping glasses, to cure with almost invariable certainty and great rapidity Alms-house ophthalmia, the different forms of which have now been considered.

There is however another form of ophthalmia, the purely scrofulous form, to which we may allude. This ophthalmia occurs more frequently in other asylums, and in private families, than in Alms-houses; but still such cases were sometimes admitted, and so varied are their forms that they scarcely admit of classification. The writer attended a case of this kind in consultation with Dr. Sargeant of the Deaf and Dumb Asylum; as the Dr. related, it commenced with several opaque spots on, or behind the cornea, in a girl of 15, who had not menstruated; at the same time he observed a slight pink circle around the cornea, and some, but no great dread of light. The Dr. treated the case antiphlogistically, and continued this treatment for three weeks, when he consulted the writer. At this time there appeared from fifteen to thirty circular opacities, from a line to two lines in diameter, around in front of each pupil. These spots on a closer examination were found to be behind the cornea, probably on the membrane of the aqueous humour, and not sufficient to obstruct the light entirely, but to render vision very imperfect. At the same time the iris remained free, but the pink circle around the cornea was distinct. We directed the patient immediately a nutritious diet with wine, which we continued till the sensibility to light had increased, and the eye became more painful. We then opened the temporal artery, and bled freely, gave Lugol's solution of iodine, in which we afterward put corrosive sublimate, (which is precipitated when put in this solution in the form of an iodine or hydriodate,) in such a proportion as to give one-fourth of a grain four times a day, with two grains of hydriodate of potash, and one of iodine, and continued the nutritious diet. After a time the sensibility to light again increased, together with the pink colour around the cornea, the opacities remaining still the same; we again opened the temporal artery, still the opacities were stationary, yet her general health was improved. We now put her on a course of phosphorus, with the syrup of sarsaparilla, to which while being made, a large quantity of yellow dock root had been added, and continued it for three months, occasionally applying a blister over the forehead or between the shoulders. Under this treatment we had the pleasure of seeing the opacities gradually disappear, and the patient

* We have been always under the impression that the vascularity of the cornea was attributable entirely to the mechanical irritation, caused by the granulations, except where a general diathesis, lymphatic or other, complicated the disease, and to such cases the writer does not at present refer. The removal of the granulations, which is effected completely by the application of the sulphate of copper, is followed by a subsidence and disappearance of the keratitis; unless a trichiasis (no unusual occurrence) still remains to keep up the irritation of the cornea. In reference to the propriety of dividing the vessels of the conjunctiva *in all* ulcerations of the cornea, we cannot agree with the writer.—*Eds. Med. Ex.*

recover. She menstruated while under treatment, and whether the treatment produced menstruation, and menstruation promoted the cure, the writer will not decide, but the cure was complete.

A man aged 28, a rock-blower by profession, was admitted totally blind. Some years previous he had received a blow on the forehead which denuded the bone over the left orbit, from the external angle to the nose. From this left eye he had not seen since the accident; this eye appeared amaurotic. From the right eye he had not seen in three weeks; this did not appear to be disorganized. A blister was applied over the forehead, and phosphorus administered. After a week the blister was repeated, and the phosphorus continued; in ten days he began to see occasional flashes of light, and in six weeks he was discharged, seeing as well as he had ever done with the right eye.

These are isolated cases, showing the probable benefit to be derived from phosphorus when the capillary system requires to be excited. This remedy has been said to act on the nervous system, but the writer has not observed such action, except on the sixth sense, through which it may stimulate the brain. Of its use in ulcerated cornea, recommendation has already been given. In these cases, after the vessels have been divided which carry red blood to the ulcers, perfect reliance can be placed on phosphorus for the remainder of the cure.

The writer has given phosphorus in many cases, and frequently with great benefit. It excites powerfully the generative system, and renders the lymph when poured out susceptible of quick organization. It probably affects the blood therefore, as much as the capillary system.

When it is decided to give phosphorus, the solution in oil should be made by putting four drachms of phosphorus in eight ounces of olive oil in a Florence flask well corked, and be heated gently in a basin of warm water, till the phosphorous melts beneath the oil, and then well shaken. This is to be repeated until the phosphorus be nearly all taken up, and then set by to cool, and afterward decanted and put into a bottle with a glass stopper. It may be given in doses of from five to twenty drops three times a day. It should not, however, be long kept, as by some action which the writer does not understand, the phosphorous loses its strength, and the physician is disapppointed. When thus given in full doses, if the dose be too large, the most common effect is a tendency to faint, while the pulse remains not materially affected or rather slow, or a slight pain in the stomach, with general heat of skin, while the pulse remains still not affected; and when in doses to produce the effect desired, it should be continued till it produces a general glow of redness on the skin, which by gentle friction shall become rose coloured. This effect should be expected, after taking it about thirty days, when, if improvement does not commence, it will be useless to continue the remedy farther.

To return to scrofulous ophthalmia. The most common form is characterized by a constantly increased sensibility to light, occasionally augmented in the morning, and diminished toward evening; while at the same period of this periodical exacerbation a pink circle will be seen on the sclerotica, surrounding the cornea. There will be an abundant flow of tears, frequent crops of pustules at the roots of the eyelashes, and a gummy secretion, probably from the glands of Meiboměus, sufficient to cause the lids to adhere in the morning. These periods of periodical exacerbation commonly occur in the spring and autumn. They are always worse in the spring, and endure from six weeks to two months, when they cease, leaving the eye more or less divested of eyelashes, reddened along the lachrymal groove, incapable of much application, and too moist for distinct vision. Now this form is hereditary, and depends on an original deficiency in the power of formation during gestation, and has at each period of exacerbation, as said above, the morning sickness and afternoon excitement of the mother. It cannot therefore be cured, but can be so far improved, that the difference of strength between such an eye, and one perfectly formed, can scarcely be observed; yet the case requires a long course and much perseverance, as well on the part of the patient as the physician. For not till much of the material composition of the eye has been absorbed and reproduced under treatment, can the physician expect to find his patient materially improved.

The writer begins with the syrup of sarsaparilla of the best kind, in doses of a table spoonfull three times a day for a child, and after two months, adds Lugol's solution of iodine, and continues these yet for two months. If at the end of four months the eye shows a marked improvement, and the pustules are reproduced less frequently, he makes an ointment of Peruvian balsam, two drachms to the ounce of lard, and with this anoints the lids every night on going to bed; he then adds to Lugol's solution minute doses of corrosive sublimate (which should be decomposed by the solution before being given) and continues for still two months; all the time giving a nutritious diet, and removing the excess of action, if there be any, by blisters between the shoulders, or a seton in the arm, and anticipates at the end of six months, something like a cure. But if there still be no improvement, he omits the iodine and corrosive sublimate, and gives phosphorus. From this remedy, in some cases, he expects the action will be too high at first, for which the temporal artery may require to be opened. After this he leaves off the phosphorus, and recurs to the iodine; and so alternating with phosphorus or iodine, while the syrup of sarsaparilla is in constant requisition. He continues for a year or twenty months, when he has never yet failed to have the satisfaction of seeing such eyes, if not perfectly cured, well enough for all ordinary purposes of life, and in his experience they have rarely *relapsed.*

---

*M. Guérin on Rachitis.*—An elaborate memoir on the general characters of Rachitis has been translated for the January number of the Western Journal of Medicine and Surgery, from the French of M. Guérin, by Dr. Colescott, of Cincinnati. We extract the general summary and conclusions of this paper.

The researches contained in this memoir may be recapitulated as follows:

1. Rachitis is a general affection of infancy, characterized by the alteration or perversion, and also the suspension of the process of developement and of reparation of the organism, and particularly of the osseous system.

2. The progress of rachitis, considered as an affection of the skeleton, comprises three distinct periods: first, the period of *incubation* or of *effusion*; second, that of *deformity;* and third, that of *resolution* or *eburnation;* to each of these periods correspond some peculiar general symptoms, and some peculiar alterations of the osseous tissue.

3. The influence of rachitis upon the osseous tissue is revealed by four distinct orders of facts: first, the *deformity;* second, the *alteration of tissue;* third, the *arrest of development;* and fourth, the *delay of ossification.*

4. The rachitic deformity of the skeleton is developed successively from below upwards, from the bones of the leg to the thigh bones, from these to the pelvis; then come successively or simultaneously the different portions of the superior limbs, the thorax, and, in the last place, the vertebral column and the cranium.

The degree of the deformities is in relation with their order of development; whence it follows that the rachitic deformity of one portion of the skeleton always implies that of the portions situated below.

5. Most of the bones of the rachitic skeleton are always relatively less developed in length and breadth than those of the healthy skeleton; this shortening which is independent of that resulting from the deformities is effected according to the same laws; that is to say, successively from below upwards, and gradually from above downwards. The proportion according to which all these parts of the skeleton are reduced from below upwards is expressed by a regular series of numbers, which permits us from the dimensions of a single bone to deduce approximately that of the other portions of the skeleton.

6. The greatest reduction of the inferior compared to that of the superior members establishes between these parts relations of length, which repeat and perpetuate those of the age at which the malady is developed.

7. The shortening of the bones considered in rachitic adults is a result composed of the arrest of development of the osseous system under the direct influence of the disease, and of the consecutive slowness of its increase subsequent to the disease.

8. The texture of rachitic bones presents entirely different characters according as they are observed during the period of *incubation, deformity*, or that of *resolution;* they differ at the beginning and the end of each of these periods, and are different, lastly, according to the *degrees* and the *age* of the affection.

9. During the period of incubation an effusion of sanguinolent matter occurs in all the interstices of the osseous tissue, the cells of the spongy tissue, the medullary canal, between the periosteum and the bone, the concentric lamellæ of the diaphyses, the epiphyses and the diaphyses, the epiphysary nuclei and their cells, in the short and the flat, as well as in the long bones; in a word, in every part of the skeleton, and all the points of the osseous tissue to which the radicles of the nutrient vessels are distributed. From this effusion result the exfoliation of the parts composing the tissue, and the swelling and enlargement of the different portions of the skeleton.

10. During the second period, that of deformity, at the same time that the frame-work of the osseous tissue is losing its consistence and growing soft, the matter which continues to be deposited in all the interstices of this tissue tends to organization: it passes successively from the cellulo-vascular to the cellulo-spongy form. This newly formed matter is particularly abundant between the periosteum and bone, the medullary membrane and the canal, the periosteum and external table of the flat bones, and between the plates of the latter.

11. During the third period, that of resolution, the newly formed tissue in the long, and in some of the short and flat bones, passes into the condition of the compact tissue, and has a tendency to confound itself with the old tissue, which retains its primary hardness. This addition of a new to the ancient tissue gives a very great increase of thickness, and especially of width to some parts of the bones which had been the seat of the organization of the new spongy tissue of the preceding period.

12. In the state I have designated under the name of *rachitic consumption*, and which results from an exaggerated degree of the affection, the unfolding and the separation of the parts composing the osseous tissue, are so great that their reunion has not been effected, nor the organization of the effused matter taken place. In this condition the partitions and osseous lamellæ have continued separate, and the consistence of the primitive bone has been so much reduced that its external layer is formed sometimes by nothing more than a thin pellicle.

13. The texture of the rachitic bones in adults when the disease has been completely dissipated presents a compactness and hardness superior to that of the healthy state. In this state, which I call *rachitic eburnation*, one can no longer discern any trace of the union of the elements of the old with those of the new bone.

14. The deformities of the spine, which happen towards the age of puberty, and all those which have not been preceded by deformities of the inferior limbs are not of a rachitic nature.

15. Rachitis is an affection essentially different from *scrofula*, or from the *tuberculous* affection of the bones, as well as from every species of *softening of the bones observed in adults;* for these latter affections the denomination of *osteomalacia* must be exclusively reserved.

### HEALTH OF THE CITY.

INTERMENTS *in the City and Liberties of Philadelphia, from the* 30*th January, to the* 6*th of February*, 1841.

| Diseases. | Adults. | Children. | Diseases. | Adults. | Children. |
|---|---|---|---|---|---|
| Apoplexy, | 1 | 1 | *Brought forward*, | 40 | 39 |
| Asthma, | 1 | 0 | Infl. of bladder, | 0 | 1 |
| Caries of Spine, | 1 | 0 | —— pleura, | 1 | 0 |
| Croup, | 0 | 6 | —— peritoneum, | 1 | 0 |
| Cong. of lungs, | 2 | 1 | Intemperance, | 1 | 0 |
| —— brain, | 0 | 1 | Jaundice, | 1 | 0 |
| Consumption of | | | Measles, | 0 | 1 |
| the lungs, | 17 | 2 | Old age, | 1 | 0 |
| Convulsions, | 1 | 7 | Palsy, | 4 | 0 |
| Cyanosis, | 0 | 1 | Small pox, | 1 | 0 |
| Diarrhœa, | 0 | 1 | Still-born, | 0 | 6 |
| Dropsy, | 1 | 1 | Ulceration of | | |
| —— head, | 0 | 6 | bowels, | 1 | 0 |
| —— breast, | 1 | 0 | Unknown, | 2 | 1 |
| Disease of the | | | | — | — |
| heart, | 2 | 0 | Total, | 53 | 48 |
| —— bowels, | 0 | 1 | —— | | |
| Dysentery, | 1 | 0 | Of the above, there | | |
| Erysipelas, | 1 | 0 | were under 1 year, | | 23 |
| Fever, scarlet, | 0 | 3 | From 1 to 2 | | 7 |
| —— continued, | 1 | 0 | 2 to 5 | | 12 |
| —— congestive, | 1 | 0 | 5 to 10 | | 5 |
| —— typhus, | 1 | 0 | 10 to 15 | | 1 |
| —— typhoid, | 1 | 0 | 15 to 20 | | 1 |
| —— puerperal, | 1 | 0 | 20 to 30 | | 12 |
| Hooping-cough, | 0 | 1 | 30 to 40 | | 15 |
| Inflammation of | | | 40 to 50 | | 7 |
| the brain, | 0 | 1 | 50 to 60 | | 7 |
| —— bronchi, | 1 | 1 | 60 to 70 | | 5 |
| —— lungs, | 2 | 4 | 70 to 80 | | 3 |
| —— stomach, | 2 | 0 | 80 to 90 | | 2 |
| —— bowels, | 1 | 1 | 90 to 100 | | 1 |
| | — | — | | | —— |
| *Carried forward*, | 40 | 39 | Total, | | 101 |

In the above are included fourteen people of colour, and nine interments from the almshouse.

